# Differences in the Social Experiences of Autistic and Non‐Autistic Adolescents by Gender

**DOI:** 10.1002/aur.70118

**Published:** 2025-09-12

**Authors:** Ellie Roberts, William Mandy, Eirini Flouri

**Affiliations:** ^1^ Division of Biosciences, Medical Sciences Building University College London London UK; ^2^ Research Department of Clinical, Educational, & Health Psychology University College London London UK; ^3^ Department of Psychology and Human Development, Institute of Education University College London London UK

**Keywords:** adolescence, autism, friendship, gender, loneliness

## Abstract

Adolescence is a time of complex social and emotional development when friendships become of particular importance. Previous research has highlighted differences in the social experience of autistic and non‐autistic adolescents, as well as that of autistic girls and boys. However, no study has compared the social experiences of autistic and non‐autistic adolescents, including gender differences, in a population‐representative sample. Using data from the Millennium Cohort Study (MCS), Sweep 6, we investigated differences between autistic (girls *n* = 111, boys *n* = 387) and non‐autistic (girls *n* = 5847, boys = 5697) adolescents (mean age = 13.7 years, range = 13–15) regarding self‐reports of: (i) having close friends, (ii) time spent with friends, (iii) social support, (iv) social alienation, (v) happiness with friendships, and (vi) having a romantic partner. Autistic adolescents reported having fewer close friends and spending less time with their friends. Autistic boys felt less socially supported than non‐autistic adolescents, while autistic girls felt more socially alienated than all other groups considered in the study. After accounting for hyperactivity and emotional problems, all girls felt more socially alienated than boys. Only autistic boys were unhappier with friendships than non‐autistic children. No group differences were found regarding romantic relationships. Some social experiences of adolescents vary greatly by both gender and diagnostic status. Further research should seek to examine the impact of these differences on mental health and well‐being.

**Summary**

- Friendships become more important and complex during adolescence.
- However, experiences of friendship vary between autistic and non‐autistic adolescents and between girls and boys.
- This study was designed to find out how social experiences may differ depending on whether someone is autistic or not, and their gender.
- We found that autistic adolescents typically report less positive social experiences and that different social struggles are reported by autistic girls and boys.
- This highlights multiple ways that the social experiences of autistic girls and boys differ, which may be useful for understanding gender differences in autism presentation and developing more targeted interventions.

## 1 Introduction

Autism is a neurodevelopmental condition characterized by social communication difficulties and repetitive behaviors (American Psychiatric Association 2013). Autistic children report having fewer friends than their non‐autistic peers (Rowley et al. 2012) and that their friendships are of lower quality (Petrina et al. 2014). It has been proposed that this arises, in part, due to the double empathy problem, whereby autistic and non‐autistic individuals experience equal difficulty understanding each other (Jones et al. 2024), as both autistic children and their close friends rate their friendship to be of lower quality (Bauminger et al. 2008). Importantly, these social difficulties increase significantly across development. For example, in early childhood, autistic children do not report being lonelier than their non‐autistic peers (Chamberlain et al. 2007), yet autistic adolescents report higher levels of loneliness compared to non‐autistic adolescents (Whitehouse et al. 2009). This may be related to the changing nature of social interactions with age. In childhood, friendships are generally underpinned by companionship and mutual play (Gifford‐Smith and Brownell 2003). However, adolescence brings about increased complexity in friendships, with a greater emphasis on mutual emotional support and intimacy (Manchanda et al. 2023). In turn, loneliness among autistic adolescents, related to both number of close friendships and satisfaction with those friendships (Jackson et al. 2018), is strongly linked to poor mental health (Lisboa White et al. 2024). Subsequently, investigating the social experience of autistic adolescence appears to be of particular importance.

A review by Petrina et al. (2014) described the social experiences of autistic children and adolescents, including friendship number, frequency of contact, activities with friends, peer victimization, and friendship quality. However, the constituent study samples were overwhelmingly male, and subsequently, much less is known about autistic girls' experiences of friendships. For example, the observation that autistic adolescents mostly spend time playing games or sports with their friends (Bauminger and Kasari 2000) is likely driven by predominantly male samples. This is particularly important as gender differences in number of friends, activities with friends, and definitions of friendship are well‐established in the general population of young people (Rose and Rudolph 2006). While previous research has postulated that autistic girls likely behave more similarly to autistic boys than to non‐autistic girls with regard to friendship (Baron‐Cohen and Wheelwright 2003), autistic girls have been observed to stay within closer proximity to peers in free play, while autistic boys typically play alone (Dean et al. 2017). Furthermore, Head et al. (2014) found that autistic girls score significantly higher on measures of social understanding and empathy than autistic boys, despite scoring lower than non‐autistic girls. This suggests that it cannot be assumed that findings from studies investigating the social experience of autistic boys can be extrapolated to autistic girls.

While previous research has demonstrated some differences in social difficulties between autistic girls and boys, less is known about how these are subjectively experienced by the individual. The social difficulties of autistic girls may also be overlooked due to social camouflaging and masking behaviors, reported more often by autistic females than males (Cook et al. 2021). That is, autistic girls may appear to function well socially by adopting particular behaviors they observe in others during their interactions in order to appear more neurotypical, concealing their social difficulties (Dean et al. 2017). This has been postulated to contribute to the under‐recognition of the social difficulties of autistic girls and women (Hull et al. 2020). Importantly, compared to autistic boys, autistic girls appear more socially driven, similarly to non‐autistic girls (Dean et al. 2017; Head et al. 2014), who in general report greater social motivation and desire to fit in than boys (Sedgewick et al. 2019). Therefore, due to their social communication difficulties coupled with their desire for friendships and belonging, autistic girls may face distinctive social challenges, which may also be difficult to identify through objective measures alone because of masking.

In addition to previous studies relying predominantly on male samples, much research considering autism, gender, and social relationships has been qualitative, based on very small samples. In addition, many studies have drawn participants from a single school (Petrina et al. 2014), which may cause issues in generalizability due to selection. Existing studies have also tended to base evaluations of the social experiences of autistic children on parent‐reported observations such as number of friends, activities with friends, duration of friendships, and frequency of meetings (Bauminger and Shulman 2003; Lyons et al. 2011), which may not accurately reflect the subjective experience of the autistic child. Kuo et al. (2013) found differences in social experiences reported by autistic girls and boys, yet it is unknown how this compares to the experiences of their non‐autistic peers. Similarly, other studies have investigated how the social experience of autistic children and adolescents compares to that of their non‐autistic peers (Kasari et al. 2011; Locke et al. 2010; Whitehouse et al. 2009) but have not examined the influence of gender. Sedgewick et al. (2019) examined whether social experiences of adolescents were influenced by gender and/or diagnostic status, with samples of autistic girls (*N* = 27) and autistic boys (*N* = 26); however, evaluations of social experiences were limited to best‐friendship quality, peer victimization, and parent‐reported social responsiveness, although further detail was obtained through semi‐structured interviews.

### 1.1 The Present Study

While several studies have examined gender differences in the social experiences of autistic children and adolescents, they are limited by the lack of a comparison with non‐autistic individuals' social experiences, as well as small sample sizes and limited measures of social experience. With one exception (Sedgewick et al. 2019), previous research investigating adolescent friendships has typically focused on differences by gender or diagnostic status but not both. No other study has made direct comparisons between all groups based on gender and diagnostic status, that is, autistic girls, non‐autistic girls, autistic boys, and non‐autistic boys. Furthermore, the existing studies have limited measures of social experience, typically focusing on number of friends, time spent with friends, and friend satisfaction (Bauminger‐Zviely et al. 2014; Kuo et al. 2013). They also lack representativeness with regard to gender, ethnicity, and location, in addition to relying on small sample sizes. Extending the existing literature by addressing these limitations, the current study used data from a large longitudinal UK birth cohort, the Millennium Cohort Study (MCS), to compare the social experiences of adolescents by diagnostic status and gender, investigating potential interactions between the two and conducting individual comparisons between all possible groups. MCS comprised a large sample with a relatively equal gender divide as well as overrepresentation of ethnic minority populations and people living in disadvantaged areas, compared to the UK as a whole. Contrary to existing research, evaluation of social experiences was varied, using measures such as having close friends, time spent with friends, and being bullied, as well as those of happiness in friendships, feeling socially supported, and social alienation, but also having a romantic partner, which research on gender differences among autistic adolescents has yet to investigate. This enabled a comprehensive examination of social experiences and how they may relate to one another. Through comparing multiple social outcomes by both gender and diagnostic status, specific differences were able to be observed between particular groups that may otherwise have been overlooked.

## 2 Methods

### 2.1 Participants

The Millennium Cohort Study (MCS) tracks the development of approximately 19,000 participants born during 2000–2002 in England, Scotland, Wales, and Northern Ireland (Joshi and Fitzsimons 2016). The MCS sample design was determined by UK electoral wards (Plewis et al. 2004) to ensure that the sample overrepresented families in high child poverty areas, as well as areas with high rates of ethnic minority populations in England. For each wave (“sweep”), ethical approval was given by UK Multi‐Centre Ethics Committees. Informed consent was given by parents before any interviews were conducted. Data are available for MCS sweeps at age 9 months (Sweep 1), 3 years (Sweep 2), 5 years (Sweep 3), 7 years (Sweep 4), 11 years (Sweep 5), 14 years (Sweep 6), and 17 years (Sweep 7). We used data from Sweep 6, when MCS first measured adolescent social experience comprehensively. At Sweep 6, 11,714 families took part in MCS. Our analytic sample included those with any data on social experience at that sweep (*N* = 11,482).

### 2.2 Measures

#### 2.2.1 Autism

The presence of an autism diagnosis was assessed at ages 5 (Sweep 3), 7 (Sweep 4), 11 (Sweep 5), and 14 (Sweep 6) through a single question asking the mother whether a doctor had ever diagnosed the cohort child with autism. Previous verifications have established parent‐reported clinician autism diagnoses to be valid (Daniels et al. 2012). Due to inconsistencies in the reporting of a diagnosis across sweeps, our autistic sample comprises those who responded “yes” at least once and never “no” after having previously responded “yes” (Mandy et al. 2022).

#### 2.2.2 Social Experience

Multiple questions investigating social experiences were drawn from Sweep 6, self‐reported by the adolescent. These addressed social support, communicating worries, having close friends, time spent with friends, feeling lonely, feeling unloved, experiencing bullying, experiencing bullying online, having a romantic partner, and friend satisfaction (see Table S1 for more details).

#### 2.2.3 Mental Health and IQ

Mental health was assessed at Sweep 6 using the hyperactivity and emotional problems subscales of the parent‐reported Strengths and Difficulties Questionnaire (SDQ). SDQ scores are a valid measure of psychopathology, with the odds of a clinical diagnosis increasing at a constant rate as SDQ scores increase (Goodman and Goodman 2009). The SDQ has also demonstrated validity in autistic samples specifically (Simonoff et al. 2013).

Verbal ability was measured at Sweep 6 with the Word Activity task which assessed participants' vocabulary and understanding of word meaning and was used as an approximation of IQ. This task has demonstrated good psychometric properties and is highly correlated with measures of verbal intelligence. Similar measures of verbal ability and vocabulary have displayed strong correlations with general intelligence (Smith et al. 2005). Associations between language ability and non‐verbal intelligence have also been observed in a sample of autistic children (Faerman et al. 2023).

### 2.3 Analytic Strategy

Analyses were conducted in STATA version 18. Descriptive statistics for all variables were used to summarize sample characteristics. Exploratory factor analysis (EFA) with promax rotation identified underlying dimensions within the social experience questions. Two‐way ANOVAs were used to determine whether autism, gender, or their interaction predicts social experience outcomes, including those identified through EFA. Tukey's test was then used to conduct all pairwise comparisons between social experience outcomes for autistic girls, autistic boys, non‐autistic girls, and non‐autistic boys.

The unadjusted model included only MCS sampling “stratum,” measured at Sweep 1 to record the type of area (ward) from which the families were sampled. This describes the nine sub‐groups of MCS families, as sampled from “advantaged” and “disadvantaged” areas across all four UK countries (England, Wales, Scotland, and Northern Ireland) and from areas of higher ethnic minority density in England. Stratum was adjusted for in all models.

The adjusted model additionally accounted for confounders (all measured at Sweep 6) including ethnicity, parental education, family structure, and sibship size. Ethnicity was recorded via the UK Census classification: White, Black Caribbean, Black African, Indian, Pakistani, Bangladeshi, Mixed, or Other Ethnic Group (including Chinese or Other). Parental education was measured as the highest educational qualification obtained by the parental respondent and based on the UK's National Vocational Qualifications classification, ranging from none (1) to the highest level (6), by Sweep 6. Family structure was the number of parents present in the household (one or two). The number of siblings was an open‐ended question. The supplementary adjusted model added as additional controls verbal ability and mental health, also measured at Sweep 6.

## 3 Results

### 3.1 Descriptive Statistics

Descriptive statistics for the sample are displayed in Tables 1 and 2. As mentioned, all social experience variables and confounders were measured in Sweep 6 at age 14 years. The sample, predominantly of white ethnicity (reflecting UK demographics [Office for National Statistics 2022)], was relatively equally divided by sex. The majority of participants were living in two‐parent households. The most common level of parental educational attainment was equivalent to NVQ Level 4 (certificate of higher education). The overall sample at age 14 appeared to report relatively positive social experiences of having close friends, seeing friends often, telling others their worries, feeling socially supported, having someone to turn to, and feeling close to others. Similarly, the majority of the sample reported that they were never bullied and did not feel lonely or unloved. Rates of friend satisfaction were relatively high. Most of the sample did not have a boyfriend or girlfriend.

**TABLE 1:** Descriptive statistics of sample characteristics (unweighted data).

Categorical variables		*N* (%)
Autism diagnosis		631 (2.1)
Sex	Female	5916 (49.9)
	Male	5936 (50.1)
Ethnicity	White	9098 (79.5)
	Mixed	543 (4.7)
	Indian	309 (2.7)
	Pakistani	584 (5.1)
	Bangladeshi	254 (2.2)
	Black Caribbean	120 (1.1)
	Black African	227 (2.0)
	Other Ethnic Group	314 (2.7)
Family structure	Two parents	8826 (75.4)
	Single parent	2884 (24.6)
Highest parental education	National Vocational Qualification (NVQ) Level 1	1203 (6.3)
	NVQ Level 2	4696 (24.4)
	NVQ Level 3	2668 (13.9)
	NVQ Level 4	6379 (33.2)
	NVQ Level 5	2295 (11.9)
Stratum	England—Advantaged	4615 (24.9)
	England—Disadvantaged	4515 (24.4)
	England—Ethnic	2393 (12.9)
	Wales—Advantaged	831 (4.5)
	Wales—Disadvantaged	1926 (10.4)
	Scotland—Advantaged	1144 (6.2)
	Scotland—Disadvantaged	1188 (6.4)
	N. Ireland—Advantaged	721 (3.9)
	N. Ireland—Disadvantaged	1199 (6.5)

Continuous variables		*N*	Mean (SD), range
Number of siblings	0	1613	1.55 (1.16), 0–10
	1	5135	
	2+	4963	
IQ (Verbal ability)		10,900	7.06 (2.63), 0–19
SDQ Hyperactivity		11,449	2.99 (2.40), 0–10
SDQ Emotional Problems		11,454	2.05 (2.14), 0–10

**TABLE 2:** Descriptive statistics of social experience variables at 14 years (unweighted data).

Categorical variables		*N* (%)
Lacks close friends	Yes	345 (3.0)
	No	11,120 (97.0)
How often sees friends	Most days	4150 (37.4)
	At least once a week	3856 (34.7)
	At least once a month	1937 (17.4)
	Less than once a month	774 (7.0)
	Never	388 (3.5)
Keeps worries to self	Yes	2826 (25.1)
	No	8441 (74.9)
Feels socially supported	Very true	9693 (85.7)
	Partly true	1557 (13.8)
	Not true at all	58 (0.5)
Has someone to turn to	Very true	8888 (78.7)
	Partly true	2112 (18.7)
	Not true at all	293 (2.6)
Doesn't feel close to anyone	Very true	259 (2.30)
	Partly true	953 (8.5)
	Not true at all	10,042 (89.2)
Has a romantic partner	Yes	1903 (16.8)
	No	9395 (83.2)
How often is bullied	Most days	482 (4.3)
	About once a week	673 (6.0)
	About once a month	565 (5.0)
	Every few months	605 (5.4)
	Less often	3117 (27.6)
	Never	5856 (51.8)
How often is bullied online	Most days	84 (0.7)
	About once a week	165 (1.5)
	About once a month	263 (2.3)
	Every few months	446 (4.0)
	Less often	2303 (20.4)
	Never	8040 (71.1)
Felt lonely	True	1031 (9.2)
	Sometimes	2827 (25.1)
	Not true	7408 (65.8)
Felt unloved	True	746 (6.6)
	Sometimes	1786 (15.9)
	Not true	8733 (77.5)

Continuous variables	*N*	Mean (SD), range
Unhappy with friends	11,287	2.07 (1.30), 1–7

### 3.2 Factor Analysis

The results from the exploratory factor analysis (EFA) with promax rotation can be seen in Table 3. EFA resulted most fittingly in a 2‐factor structure comprising “social support” and “social alienation,” determined by a cut‐off of < 0.32 for factor loadings. Social support included the item measures: “have family and friends who make me feel supported and safe,” “feel like I have someone to turn to,” “keep worries to myself” and “no one feels close.” The latter two were reverse coded prior to factor analysis. Social alienation contained the two measures of bullying in Sweep 6: “often picked on/hurt by others” and “bullied online,” as well as the two items “felt lonely” and “felt nobody really loved me.” The two bullying measures were reverse coded prior to factor analysis, as higher scores originally represented less frequent victimization. Time spent with friends was not included in the factor analysis due to collinearity with lacking close friends and factor results were identical with the inclusion of either variable. Lacking close friends, not having a romantic partner, and being unhappy with friends did not load successfully onto any factor but likely represented distinct social measures. Therefore, we investigated each of these as distinct outcomes in subsequent analyses.

**TABLE 3:** Factor loadings from exploratory factor analysis of the social experience variables after promax rotation.

Variable	Factor 1 (social support)	Factor 2 (social alienation)
Lacks close friends	0.2517	−0.0220
Tells others worries	**0.4895**	0.0131
Feels safe & supported	**0.5547**	0.0842
Has someone to turn to	**0.7229**	−0.1300
Feels close to others	**0.3796**	0.1133
No romantic partner	0.1140	−0.1830
Often bullied	−0.0338	**0.4376**
Often bullied online	0.0247	**0.4563**
Unhappy with friends	0.2580	0.2720
Feels lonely	0.0656	**0.8665**
Feels unloved	0.0129	**0.7908**

### 3.3 Univariable Analysis of Social Experiences by Group

Mean differences in the social experiences of non‐autistic (NT) girls, non‐autistic boys, autistic girls, and autistic boys can be seen in Table 4. All variables demonstrated significant group differences except for having a romantic partner. Group comparisons for individual factor items can be found in Table S2.

**TABLE 4:** Comparison of social experiences by group (unweighted data).

Variable (Prob>*F*)	NT girls (*N* = 5847)		NT boys (*N* = 5697)		Autistic girls (*N* = 111)		Autistic boys (*N* = 387)	
	Mean 95% CI	SD	Mean 95% CI	SD	Mean 95% CI	SD	Mean 95% CI	SD
Social support***	−0.010 −0.033, 0.013 *N* = 5550	0.868	0.030 0.009, 0.052 *N* = 5180	0.790	−0.281 −0.494, −0.068 *N* = 76	0.932	−0.325 −0.447, −0.203 *N* = 250	0.979
Social alienation***	0.180 0.15, 0.21 *N* = 5550	1.011	−0.213 −0.23, −0.19 *N* = 5180	0.730	0.824 0.58, 1.07 *N* = 76	1.08	0.159 0.05, 0.27 *N* = 250	0.888
Lacks close friends***	1.020 1.02, 1.02 *N* = 5677	0.140	1.035 1.03, 1.04 *N* = 5400	0.183	1.120 1.05, 1.19 *N* = 83	0.328	1.111 1.08, 1.15 *N* = 305	0.315
Doesn't often see friends***	2.060 2.03, 2.09 *N* = 5560	1.053	2.003 1.97, 2.03 *N* = 5203	1.060	2.425 2.12, 2.73 *N* = 73	1.301	2.461 2.30, 2.62 *N* = 269	1.314
Doesn't have romantic partner	1.835 1.82, 1.85 *N* = 5639	0.371	1.829 1.82, 1.84 *N* = 5318	0.376	1.769 1.67, 1.87 *N* = 78	0.424	1.814 1.77, 1.86 *N* = 263	0.390
Unhappy with friends***	2.149 2.11, 2.18 *N* = 5634	1.356	1.953 1.92, 1.99 *N* = 5311	1.211	2.597 2.21, 2.98 *N* = 77	1.688	2.411 2.23, 2.59 *N* = 265	1.498

*Note*: *p < 0.05; **p < 0.01; ***p < 0.001.

### 3.4 Multivariable Analysis of Social Experiences by Group

Two‐way ANOVAs and Tukey's tests were then conducted for each social experience variable, before and after adjustment for confounders, as seen in Table 5. ‘Predictors’ in these ANOVAs were autism, gender, and the interaction. Results for the second adjusted model can be seen in the Table S3. Interactions between gender and autism were tested but were not found to be significant.

**TABLE 5:** Results of two‐way ANOVAs & Tukey's tests of the role of autism, gender and their interaction in social experiences, before and after confounder adjustment.

Social support factor					
Unadjusted (*N* = 8,264)	Partial SS	df		*F*	Prob>*F*
Autism diagnosis***	11.247	1		16.44	0.0001
Gender (Female)	0.000	1		0.00	0.9882
Autism * Gender	0.199	1		0.29	0.5896

Adjusted (*N* = 7,916)					
Autism diagnosis***	8.637	1		12.86	0.0003
Gender (Female)	0.030	1		0.05	0.8318
Autism * Gender	0.060	1		0.09	0.7658

Comparison	Contrast	SE	*t*	*P*>[*t*]	95% CI
NT Girls vs NT Boys	0.034	0.018	1.83	0.259	−0.014, 0.081
Autistic Boys vs NT Boys***	0.296	0.063	4.67	0.000	0.133, 0.460
Autistic Girls vs NT Boys	0.261	0.112	2.32	0.093	−0.028, 0.549
Autistic Boys vs NT Girls***	0.263	0.063	4.14	0.000	0.100, 0.426
Autistic Girls vs NT Girls	0.227	0.112	2.02	0.181	−0.062, 0.516
Autistic Girls vs Autistic Boys	−0.036	0.128	−0.28	0.992	−0.364, 0.292

Adjusted					
NT Girls vs NT Boys	0.031	0.019	1.67	0.338	−0.017, 0.079
Autistic Boys vs NT Boys***	0.268	0.064	4.18	0.000	0.103, 0.432
Autistic Girls vs NT Boys	0.247	0.113	2.19	0.125	−0.042, 0.536
Autistic Boys vs NT Girls***	0.237	0.064	3.70	0.001	0.072, 0.401
Autistic Girls vs NT Girls	0.216	0.113	1.92	0.222	−0.074, 0.505
Autistic Girls vs Autistic Boys	−0.021	0.128	−0.16	0.998	−0.350, 0.308

Social alienation factor					
Unadjusted (*N* = 8,264)	Partial SS	df		*F*	Prob>*F*
Autism diagnosis***	26.322	1		33.73	0.0000
Gender (Female)***	41.017	1		52.56	0.0000
Autism * Gender	2.151	1		2.76	0.0969

Adjusted (N = 7,916)					
Autism diagnosis***	22.642	1		29.14	0.0000
Gender (Female)***	37.834	1		48.70	0.0000
Autism * Gender	1.823	1		2.35	0.1256

Comparison	Contrast	SE	*t*	*P*>[*t*]	95% CI
NT Girls vs NT Boys***	0.385	0.020	19.51	0.000	0.334, 0.436
Autistic Boys vs NT Boys***	0.286	0.068	4.22	0.000	0.112, 0.460
Autistic Girls vs NT Boys***	0.900	0.120	7.50	0.000	0.591, 1.208
Autistic Boys vs NT Girls	−0.099	0.068	−1.46	0.462	−0.273, 0.075
Autistic Girls vs NT Girls***	0.515	0.120	4.29	0.000	0.206, 0.823
Autistic Girls vs Autistic Boys***	0.614	0.136	4.50	0.000	0.263, 0.964

Adjusted					
NT Girls vs NT Boys***	0.382	0.020	18.97	0.000	0.330, 0.434
Autistic Boys vs NT Boys***	0.272	0.070	3.91	0.001	0.094, 0.451
Autistic Girls vs NT Boys***	0.870	0.122	7.12	0.000	0.556, 1.183
Autistic Boys vs NT Girls	−0.110	0.070	−1.58	0.392	−0.288, 0.069
Autistic Girls vs NT Girls***	0.487	0.122	3.99	0.000	0.174, 0.801
Autistic Girls vs Autistic Boys***	0.597	0.139	4.30	0.000	0.240, 0.954

Lacks close friends					
Unadjusted (*N* = 8,557)	Partial SS	df		*F*	Prob>*F*
Autism diagnosis***	1.250	1		44.71	0.0000
Gender (Female)	0.047	1		1.68	0.1944
Autism * Gender	0.003	1		0.10	0.7491

Adjusted (N = 8,183)					
Autism diagnosis***	1.252	1		45.79	0.0000
Gender (Female)	0.033	1		1.20	0.2726
Autism * Gender	0.002	1		0.06	0.8133

Comparison	Contrast	SE	*t*	*P*>[*t*]	95% CI
NT Girls vs NT Boys**	−0.012	0.004	−3.28	0.006	−0.022, −0.003
Autistic Boys vs NT Boys***	0.086	0.012	7.41	0.000	0.057, 0.116
Autistic Girls vs NT Boys*	0.067	0.022	3.06	0.012	0.010, 0.122
Autistic Boys vs NT Girls***	0.099	0.012	8.46	0.000	0.069, 0.128
Autistic Girls vs NT Girls**	0.079	0.022	3.61	0.002	0.023, 0.134
Autistic Girls vs Autistic Boys	−0.020	0.024	−0.82	0.846	−0.083, 0.043

Adjusted					
NT Girls vs NT Boys*	−0.011	0.003	−2.90	0.020	−0.020, −0.001
Autistic Boys vs NT Boys***	0.088	0.012	7.38	0.000	0.057, 0.119
Autistic Girls vs NT Boys**	0.071	0.022	3.23	0.007	0.015, 0.128
Autistic Boys vs NT Girls***	0.099	0.012	8.29	0.000	0.068, 0.129
Autistic Girls vs NT Girls***	0.082	0.022	3.72	0.001	0.025, 0.139
Autistic Girls vs Autistic Boys	−0.017	0.025	−0.67	0.907	−0.080, 0.047

Doesn’t spend much time with friends					
Unadjusted (*N* = 8,297)	Partial SS	df		*F*	Prob>*F*
Autism diagnosis***	34.106	1		31.60	0.0000
Gender (Female)	0.021	1		0.02	0.8898
Autism * Gender	0.867	1		0.80	0.3701

Adjusted (*N* = 7,943)					
Autism diagnosis***	43.866	1		42.15	0.0000
Gender (Female)	0.235	1		0.23	0.6346
Autism * Gender	1.492	1		1.43	0.2311

Comparison	Contrast	SE	*t*	*P*>[*t*]	95% CI
NT Girls vs NT Boys*	0.061	0.023	2.65	0.040	0.002, 0.121
Autistic Boys vs NT Boys***	0.528	0.077	6.83	0.000	0.329, 0.726
Autistic Girls vs NT Boys**	0.444	0.142	3.12	0.010	0.078, 0.810
Autistic Boys vs NT Girls***	0.466	0.077	6.05	0.000	0.268, 0.665
Autistic Girls vs NT Girls*	0.383	0.142	2.69	0.036	0.017, 0.748
Autistic Girls vs Autistic Boys	−0.084	0.160	−0.52	0.954	−0.496, 0.328

Adjusted					
NT Girls vs NT Boys	0.059	0.023	2.54	0.054	−0.001, 0.119
Autistic Boys vs NT Boys***	0.631	0.079	8.03	0.000	0.429, 0.833
Autistic Girls vs NT Boys**	0.494	0.144	3.43	0.003	0.124, 0.865
Autistic Boys vs NT Girls***	0.572	0.079	7.29	0.000	0.370, 0.774
Autistic Girls vs NT Girls*	0.435	0.144	3.02	0.013	0.065, 0.805
Autistic Girls vs Autistic Boys	−0.137	0.162	−0.85	0.833	−0.554, 0.280

Doesn’t have romantic partner					
Unadjusted (*N* = 8,439)	Partial SS	df		*F*	Prob>*F*
Autism diagnosis	0.007	1		0.05	0.8148
Gender (Female)	0.036	1		0.27	0.6057
Autism * Gender	0.102	1		0.75	0.3869

Adjusted (*N* = 8,071)					
Autism diagnosis	0.005	1		0.04	0.8505
Gender (Female)	0.014	1		0.10	0.7502
Autism * Gender	0.069	1		0.51	0.4752
Comparison	Contrast	SE	*t*	*P*>[*t*]	95% CI
NT Girls vs NT Boys	0.010	0.008	1.21	0.618	−0.011, 0.031
Autistic Boys vs NT Boys	0.018	0.027	0.65	0.915	−0.053, 0.088
Autistic Girls vs NT Boys	−0.021	0.050	−0.43	0.974	−0.149, 0.106
Autistic Boys vs NT Girls	0.008	0.027	0.29	0.991	−0.063, 0.078
Autistic Girls vs NT Girls	−0.031	0.050	−0.63	0.923	−0.159, 0.096
Autistic Girls vs Autistic Boys	−0.039	0.056	−0.70	0.898	−0.183, 0.105

Adjusted					
NT Girls vs NT Boys	0.011	0.008	1.37	0.517	−0.010, 0.033
Autistic Boys vs NT Boys	0.026	0.028	0.92	0.793	−0.047, 0.099
Autistic Girls vs NT Boys	−0.004	0.051	−0.07	1.000	−0.134, 0.126
Autistic Boys vs NT Girls	0.015	0.028	0.52	0.955	−0.058, 0.087
Autistic Girls vs NT Girls	−0.015	0.050	−0.30	0.991	−0.145, 0.115
Autistic Girls vs Autistic Boys	−0.030	0.057	−0.52	0.954	−0.177, 0.117

Unhappy with friends					
Unadjusted (*N* = 8,434)	Partial SS	df		*F*	Prob>*F*
Autism diagnosis***	22.427	1		13.55	0.0002
Gender (Female)	2.442	1		1.48	0.2244
Autism * Gender	0.646	1		0.39	0.5321
Adjusted (*N* = 8,071)					
Autism diagnosis**	15.805	1		9.65	0.0019
Gender (Female)	2.174	1		1.33	0.2492
Autism * Gender	0.616	1		0.38	0.5398
Comparison	Contrast	SE	*t*	*P*>[*t*]	95% CI
NT Girls vs NT Boys***	0.182	0.028	6.38	0.000	0.109, 0.255
Autistic Boys vs NT Boys***	0.426	0.095	4.47	0.000	0.181, 0.670
Autistic Girls vs NT Boys*	0.484	0.173	2.79	0.027	0.039, 0.929
Autistic Boys vs NT Girls	0.244	0.095	2.57	0.051	−0.000, 0.488
Autistic Girls vs NT Girls	0.302	0.173	1.75	0.300	−0.143, 0.747
Autistic Girls vs Autistic Boys	0.058	0.195	0.30	0.911	−0.444, 0.560

Adjusted					
NT Girls vs NT Boys***	0.177	0.029	6.11	0.000	0.103, 0.252
Autistic Boys vs NT Boys***	0.374	0.098	3.83	0.001	0.123, 0.625
Autistic Girls vs NT Boys	0.428	0.176	2.44	0.070	−0.023, 0.879
Autistic Boys vs NT Girls	0.197	0.097	2.02	0.180	−0.053, 0.447
Autistic Girls vs NT Girls	0.251	0.176	1.43	0.481	−0.200, 0.702
Autistic Girls vs Autistic Boys	0.054	0.198	0.27	0.993	−0.456, 0.564

*Note*: variables adjusted for include ethnicity, parental education, family structure and sibship size. *p < 0.05; **p < 0.01; ***p < 0.001.

#### 3.4.1 Social Support

Perceived social support was lower for autistic individuals compared to those without an autism diagnosis. Pairwise comparisons revealed that autistic boys felt less social support than non‐autistic boys and non‐autistic girls. No differences were observed between autistic boys and autistic girls, non‐autistic boys and non‐autistic girls, or autistic girls and non‐autistic boys or non‐autistic girls.

#### 3.4.2 Social Alienation

Feelings of social alienation were higher for girls compared to boys and for autistic compared to non‐autistic individuals. Non‐autistic boys did not feel as socially alienated as non‐autistic girls or autistic boys. Notably, autistic girls felt more socially alienated than non‐autistic girls, non‐autistic boys, and autistic boys. Non‐autistic girls felt more socially alienated than autistic boys when the effects of hyperactivity and emotional problems were considered (see Table S3). Feelings of social alienation did not differ between autistic boys and non‐autistic girls.

#### 3.4.3 Friendships

Autism was a significant predictor of lacking close friends. Autistic boys reported lacking close friends more than non‐autistic boys and non‐autistic girls. Similarly, autistic girls lacked close friends more than non‐autistic boys and non‐autistic girls. Non‐autistic boys also lacked close friends compared to non‐autistic girls. Autism was also a significant predictor of not spending much time with friends. Autistic girls spent less time with friends compared to non‐autistic girls and non‐autistic boys. Autistic boys also spent less time with friends compared to non‐autistic boys and non‐autistic girls. Finally, autism was a significant predictor of being more unhappy with friends. Non‐autistic boys were happier with their friends compared to autistic boys and non‐autistic girls. Autistic girls and autistic boys did not differ in lacking close friends or not spending much time with friends. Non‐autistic girls and non‐autistic boys also did not differ in not spending much time with friends.

#### 3.4.4 Romantic Partners

No group differences were observed in having a romantic partner, either before or after adjustment.

## 4 Discussion

This is the first study using a population‐representative cohort to compare the social experiences of autistic and non‐autistic adolescents, while also examining the role of gender. Using data from MCS, it examined the social experiences of 14‐year‐old UK adolescents in relation to diagnostic status, gender, and their interaction. Differences were observed in the experiences of autistic and non‐autistic adolescents, autistic girls and boys, and non‐autistic girls and boys. As expected, autistic adolescents reported more negative social experiences than non‐autistic adolescents. Furthermore, specific social experiences were observed with regard to gender, with some differences emerging between both autistic girls and boys and non‐autistic girls and boys.

Autistic adolescents reported lacking close friends and spending less time with friends compared to non‐autistic adolescents. Autistic boys felt less socially supported than non‐autistic adolescents and felt unhappier in their friendships and more socially alienated than non‐autistic boys. Meanwhile, autistic girls felt more socially alienated than non‐autistic adolescents (boys and girls) and autistic boys. Previous research has shown loneliness to be associated with number of close friendships and friendship satisfaction in autistic adolescents (Jackson et al. 2018). However, the current findings demonstrate that this varies by gender. The finding that autistic girls were also unhappier in their friendships compared to non‐autistic boys did not remain following adjustment for confounders. Despite both autistic boys and girls reporting lacking close friends and spending less time with their friends, they differed in happiness in friendships, social support, and social alienation.

Social experience differences also emerged between non‐autistic boys and girls, with non‐autistic girls having more close friends but higher levels of unhappiness in their friendships as well as social alienation. This highlights both the disparity between different measures of the adolescent social experience and the role of gender. It appears that girls experience more friendship difficulties than boys, but because of their greater number of friends, they may be perceived as more socially successful. Boys tend to form friendships based on shared interests and activities, often sports or video games, while girls are generally more interested in talking to one another (Rose and Rudolph 2006), which could explain why neurotypical boys reported having fewer close friends than neurotypical girls. However, female friendships demonstrate higher rates of relational aggression and instability (Chester et al. 2017; Sedgewick et al. 2019), which may contribute to the observation that non‐autistic girls were less happy with their friendships compared to non‐autistic boys. The observation that non‐autistic boys spent more time with their friends compared to non‐autistic girls did not survive adjustment for confounders.

Autistic boys but not girls reported feeling less socially supported compared to neurotypical adolescents. Due to social cognition and communication differences, autistic and non‐autistic individuals likely find it more challenging to establish supportive relationships with each other as a result of the double‐empathy problem (Bishop‐Fitzpatrick et al. 2018; Jones et al. 2024). Previous studies have shown that autistic individuals report lower levels of social support compared to the general population (Humphrey and Symes 2010), as well as finding social interactions more stressful (Hirvikoski and Blomqvist 2015). However, the results of the current study suggest that this may be specific to autistic boys. Non‐autistic boys have reported that their friendships typically revolve around recreational activities, while emotional closeness is more strongly associated with female friendships (Rudolph and Dodson 2022). Girls engage in supportive discussions more often and respond to friends' concerns in a more positive and engaged manner (Rose et al. 2016). The combined effect of diagnostic status on autistic–non‐autistic interactions and also of gender may cause autistic boys specifically to feel less socially supported than non‐autistic adolescents.

On the other hand, autistic girls reported feeling more socially alienated than non‐autistic adolescents and autistic boys. Assuming that most friendships are with others of the same sex (Rudolph and Dodson 2022), as is typical in adolescence, distinctive difficulties that arise in female friendships may contribute to the heightened social alienation of autistic girls. Autistic individuals have reported higher levels of loneliness compared to the general population (Grace et al. 2022). This may interact with gender to produce particularly strong feelings of social alienation in autistic girls. While female friendships demonstrate greater social support, relational aggression within these friendships, such as gossiping, exclusion, and controlling, is more prevalent (Putallaz et al. 2007; Sedgewick et al. 2019). Autistic girls have also reported experiencing relational aggression more frequently and finding it more difficult to manage than non‐autistic girls (Sedgewick et al. 2019). Another phenomenon that may contribute to feelings of social alienation in girls is masking behaviors (Cook et al. 2021). Despite masking typically being associated with autistic individuals, non‐autistic girls have also been found to report masking, adopting particular behaviors to fit in, to a similar degree as autistic girls (Sedgewick et al. 2019). However, this may produce a sense of emotional loneliness and lack of authentic intimacy in friendships. Although girls report having close friends and general friendship satisfaction, they experience higher levels of insecurity in their friendships (Sedgewick et al. 2019). It is likely that the effect of both diagnostic status on autistic‐non‐autistic interactions and gender is driving this excess risk of social alienation among autistic girls.

Another notable observation is the lack of an inverse relationship between social support and social alienation. Autistic boys, but not girls, do not feel as socially supported as non‐autistic adolescents. However, despite feeling less socially supported than non‐autistic adolescents, autistic boys do not feel more socially alienated than they. On the other hand, autistic girls feel more socially alienated than everyone else, but do not feel less socially supported. Similarly, non‐autistic girls have more close friends than non‐autistic boys but feel more socially alienated. This highlights social alienation as a distinctive experience that cannot be entirely defined by a lack of social support or friends. Social alienation as a factor comprises the actively negative experiences of being bullied, feeling lonely, and feeling unloved, while a lack of social support is not necessarily experienced as inherently negative. Due to the qualitatively different nature of friendships between boys and girls, this is likely more often observed within female friendships.

In the supplementary analyses, adding hyperactivity and emotional problems (as measured by the SDQ) caused differences to emerge in feelings of social alienation between non‐autistic girls and autistic boys. Hyperactivity and emotional problems correlated with increased feelings of social alienation, and as these were more prevalent in autistic boys, their presence in the analysis likely explained some of the social alienation of autistic boys. Adjusting for hyperactivity and emotional problems resulted in the finding that non‐autistic girls feel more socially alienated than autistic boys. However, the finding that autistic girls feel more socially alienated than all boys remained unchanged following adjustment for hyperactivity and emotional problems. Together, these observations demonstrate that social alienation is a more common experience for both autistic and non‐autistic girls compared to boys, and feeling more socially alienated cannot be explained by higher rates of internalizing problems in girls. This contradicts previous postulations that autistic girls share more social similarities with autistic boys than non‐autistic girls (Baron‐Cohen and Wheelwright 2003), as gender appears to be more strongly driving specific social experiences of adolescents. Autistic girls may be a special case, however. Autistic boys may be more able to engage in typical recreational activities with other boys, while autistic and non‐autistic girls may struggle to engage in the highly social nature of female friendships with each other (Sedgewick et al. 2019), in addition to autistic girls representing a rarer demographic (Loomes et al. 2017).

In adulthood, autistic females have consistently been observed to suffer from higher rates of most mental health problems compared to autistic males (Martini et al. 2022; Sedgewick et al. 2021), with specific social differences likely explaining some of this gender difference. For example, although loneliness mediates some of the effects of autistic traits on mental health across gender groups (Schiltz et al. 2021), qualitative reports from autistic women have highlighted how being bullied in school has led to developing negative perceptions of themselves and their environment, and difficulties in interpersonal relationships in adulthood (Sunagawa 2023). This study showed that even in adolescence autistic females report unique negative social experiences, particularly social alienation and friendship problems. The finding that this gender difference is present in adolescence is pertinent, as half of mental health problems develop by age 14, and 75% by age 24 (Kessler et al. 2005), and friendships are thought to promote mental health and emotional development (Manchanda et al. 2023; Mazurek 2014).

The findings of this study therefore highlight a need for gender‐informed interventions to specifically target feelings of social alienation in girls, such as relational conflict resolution. Interventions which aid the development of satisfying friendships between autistic and non‐autistic children, such as promoting understanding of the double empathy problem and the responsibility of non‐autistic, as well as autistic people, to manage the challenges of cross‐neurotype interactions (Mandy 2023), should be prioritized. The current study's use of multiple outcomes has enabled a detailed understanding of autistic adolescents' social experiences regarding how different experiences may or may not correlate, and specific differences by gender. These findings emphasize the importance for clinicians to recognize how gender may affect the social experience of autistic adolescents, and the distinctive difficulties that autistic girls and boys may experience. Subsequently, comprehensive assessments of autistic individuals' social experiences should be encouraged. For example, future studies should consider including measures of both social support and social alienation, as reports of adequate social support do not necessarily reflect a lack of experience of social alienation. These findings also highlight a need for future studies to use comprehensive measures to capture the specificity of the social experiences of autistic girls and boys.

There are numerous considerations and limitations regarding the interpretation of the results of the current study. The null findings regarding romantic relationships were slightly unexpected but may be attributed to the age of the sample, as 14 may be too young an age for differences in romantic relationships to show. The sample size was determined by those who completed at least some of the social experience measures during Sweep 6, and missing data were not accounted for, indicating that the study sample may be selective. In terms of content, further research may seek to identify when differences (if any) in romantic relationships may emerge. Importantly, due to the cross‐sectional nature of the data, the possibility of reverse causation cannot be ignored. This is particularly problematic when interpreting the additional models that accounted for mental health (hyperactivity and emotional problems), as the possibility that poor social experiences lead to emotional problems must be considered. These mental health measures are further limited by being parent‐reported. Another limitation is the lack of comprehensive approximation for IQ at Sweep 6, as the only cognitive ability measure available at that sweep was the Word Task, a measure of verbal ability and vocabulary.

There are several additional areas of interest for future research to explore. Despite this study demonstrating differences in social experiences at age 14, it is unknown at what age specifically these differences start to emerge. As these social experiences were first measured at Sweep 6, such longitudinal analysis was not possible. Further research should seek to uncover when differences are first observed to better inform early identification and intervention. Similarly, further research could monitor the progression of these social experiences into adulthood to understand whether experiences such as social alienation are particularly significant during adolescence. While this study highlights group differences in social difficulties by autism and gender, and to a large extent also their interaction, the impact of social differences for these groups was not examined. For example, research could investigate whether this produces differential impacts on mental health by autism and gender and compare how the apparently orthogonal constructs of social support and social alienation may determine wellbeing in autistic and non‐autistic boys and girls.

## 5 Conclusion

Multiple differences are observed in the social experiences of adolescents, determined by autism and gender. Autistic girls and boys report having fewer close friends and spending less time with their friends than non‐autistic adolescents. Only autistic boys feel less socially supported than non‐autistic adolescents, while autistic girls feel more socially alienated than all groups. Even after accounting for hyperactivity and emotional problems, all girls feel more socially alienated than boys. Only autistic boys report being unhappier with their friendships than non‐autistic children. No differences are observed regarding romantic relationships between gender or diagnostic status.

## Conflicts of Interest

The authors declare no conflicts of interest.

## Supporting information


**Supplementary Table 1** Social experience variables.

## Data Availability

The data that support the findings of this study are openly available in Millennium Cohort Study at http://doi.org/10.5255/UKDA‐Series‐2000031.
