# Supplementary material for: Differences in the Social Experiences of Autistic and Non‐Autistic Adolescents by Gender
Source: Autism Res. 2025 Sep 12;18(11):2307–22. doi: 10.1002/aur.70118 (PMC12661271; doi:10.1002/aur.70118)
Supplement: Supplementary file 1 — Supplementary Table 1 Social experience variables. [file AUR-18-2307-s001.docx]

Supplementary Table 1. Social experience variables

| **Outcome** | **Measure** | **Scoring** |
| --- | --- | --- |
| Social support | “I have family and friends who help me feel safe, secure and happy” “There is someone I trust whom I would turn to if I had problems”  “There is no one I feel close to” | Very true Partly true Not true at all |
| Telling others your worries | “What do you do if you are worried about something? (Keep it to myself)” | Yes No |
| Having close friends | “Do you have any close friends? (friends = other young people)” | Yes  No |
| Time spent with friends | “When not at school, how often do you spend time with your close friends?” | Most days  At least once a week  At least once a month  Less often than once a month  Never |
| Social alienation | “I felt lonely”  “I thought nobody really loved me” | Not true  Sometimes  True |
| Experiencing bullying | “How often do other children hurt or pick on you?” | Most days  About once a week  About once a month  Every few months  Less often  Never |
| Experiencing bullying online | “How often do other children bully you online?” | Most days  About once a week  About once a month  Every few months  Less often  Never |
| Having a romantic partner | “Do you have a boyfriend/girlfriend?” | Yes  No |
| Friendship satisfaction | “How happy are you with your friends?” | 1 (Completely happy) – 7 (Not at all happy) |

Supplementary Table 2. Mean outcomes for factor item variables (unweighted data)

|  | **NT Girls (N = 9,434)** | | **NT Boys (N = 9,224)** | | **Autistic Girls (N = 111)** | | **Autistic Boys (N = 387)** | |
| --- | --- | --- | --- | --- | --- | --- | --- | --- |
| **Variable (Prob>F)** | Mean | SD | Mean | SD | Mean | SD | Mean | SD |
| Keeps worries to self** | .235 N = 5,629 | .424 | .266 N = 5,295 | .442 | .269 N = 78 | .446 | .291 N = 265 | .455 |
| Doesn’t feel safe & supported*** | 1.151 N = 5,637 | .369 | 1.139 N = 5,326 | .362 | 1.231 N = 78 | .424 | 1.236 N = 267 | .467 |
| No one to turn to*** | 1.208 N = 5,634 | .458 | 1.266  N = 5,316 | .502 | 1.167 N = 78 | .408 | 1.389  N = 265 | .574 |
| Feels close to others*** | 2.859 N = 5,617 | .401 | 2.887 N = 5,296 | .388 | 2.795 N = 78 | .493 | 2.768 N = 263 | .520 |
| Not often bullied*** | 5.043  N = 5,632 | 1.381 | 5.037  N = 5,322 | 1.393 | 3.897 N = 78 | 1.991 | 4.331 N = 266 | 1.825 |
| Not often bullied online*** | 5.436 N = 5,634 | .983 | 5.676 N = 5,324 | .750 | 5.307 N = 78 | 1.166 | 5.581 N = 265 | .930 |
| Feels lonely*** | 1.582  N = 5,614 | .714 | 1.268  N = 5,309 | .537 | 1.974  N = 78 | .755 | 1.475  N = 265 | .628 |
| Feels unloved*** | 1.396  N = 5,616 | .658 | 1.172  N = 5,306 | .454 | 1.731  N = 78 | .750 | 1.325  N = 265 | .603 |

Supplementary Table 3. Results of two-way ANOVAs & Tukey’s tests of the role of autism, gender and their interaction in social experiences, after additional confounder adjustment

| **Social support factor** (N = 7,372) | | | | | |
| --- | --- | --- | --- | --- | --- |
| Autism diagnosis | .199 | 1 | | 0.30 | 0.5809 |
| Gender (Female) | .129 | 1 | | 0.20 | 0.6566 |
| Autism * Gender | .421 | 1 | | 0.64 | 0.4226 |
| **Comparison** | | | | | |
| NT Girls vs NT Boys | .022 | .020 | 1.11 | 0.683 | -.029, .074 |
| Autistic Boys vs NT Boys | .105 | .067 | 1.57 | 0.398 | -.067, .028 |
| Autistic Girls vs NT Boys | .006 | .117 | 0.05 | 1.000 | -.293, .306 |
| Autistic Boys vs NT Girls | .083 | .067 | 1.23 | 0.607 | -.090, .256 |
| Autistic Girls vs NT Girls | -.016 | .117 | -0.14 | 0.999 | -.315, .284 |
| Autistic Girls vs Autistic Boys | -.099 | .131 | -0.75 | 0.875 | -.435, .238 |
| **Social alienation factor** (N = 7,372) | | | | | |
| Autism diagnosis | 1.723 | 1 | | 2.31 | 0.1282 |
| Gender (Female)*** | 26.236 | 1 | | 35.25 | 0.0000 |
| Autism * Gender | .746 | 1 | | 1.00 | 0.3169 |
| **Comparison** | | | | | |
| NT Girls vs NT Boys*** | .354 | .021 | 16.52 | 0.000 | .299, .409 |
| Autistic Boys vs NT Boys | .045 | .072 | 0.63 | 0.922 | -.139, .229 |
| Autistic Girls vs NT Boys*** | .545 | .124 | 4.38 | 0.000 | .225, .864 |
| Autistic Boys vs NT Girls*** | -.309 | .072 | -4.30 | 0.000 | -.493, -.124 |
| Autistic Girls vs NT Girls | .191 | .124 | 1.54 | 0.415 | -.128, .510 |
| Autistic Girls vs Autistic Boys** | .499 | .140 | 3.58 | 0.002 | .141, .858 |
| **Lacks close friends** (N = 7,605) | | | | | |
| Autism diagnosis*** | .686 | 1 | | 25.68 | 0.0000 |
| Gender (Female) | .038 | 1 | | 1.41 | 0.2350 |
| Autism * Gender | .000 | 1 | | 0.00 | 0.9816 |
| **Comparison** | | | | | |
| NT Girls vs NT Boys** | -.015 | .004 | -3.77 | 0.001 | -.025, -.005 |
| Autistic Boys vs NT Boys*** | .068 | .013 | 5.36 | 0.000 | .036, .101 |
| Autistic Girls vs NT Boys | .053 | .023 | 2.27 | 0.105 | -.007, .112 |
| Autistic Boys vs NT Girls*** | .083 | .013 | 6.52 | 0.000 | .051, .116 |
| Autistic Girls vs NT Girls* | .068 | .023 | 2.93 | 0.018 | .008, .127 |
| Autistic Girls vs Autistic Boys | -.015 | .026 | -0.61 | 0.928 | -.082, .050 |
| **Doesn’t spend much time with friends** (N = 7,384) | | | | | |
| Autism diagnosis*** | 25.882 | 1 | | 25.36 | 0.0000 |
| Gender (Female) | 2.573 | 1 | | 2.52 | 0.1123 |
| Autism * Gender | 2.103 | 1 | | 2.06 | 0.1512 |
| **Comparison** | | | | | |
| NT Girls vs NT Boys | -.013 | .025 | -0.54 | 0.950 | -.078, .051 |
| Autistic Boys vs NT Boys*** | .564 | .083 | 6.76 | 0.000 | .350, .779 |
| Autistic Girls vs NT Boys | .306 | .152 | 2.02 | 0.182 | -.084, .695 |
| Autistic Boys vs NT Girls*** | .578 | .084 | 6.89 | 0.000 | .362, .793 |
| Autistic Girls vs NT Girls | .319 | .152 | 2.11 | 0.151 | -.070, .708 |
| Autistic Girls vs Autistic Boys | -.258 | .169 | -1.53 | 0.420 | -.693, .176 |
| **Doesn’t have a romantic partner** (N = 7,511) | | | | | |
| Autism diagnosis | .107 | 1 | | 0.79 | 0.3733 |
| Gender (Female) | .074 | 1 | | 0.54 | 0.4607 |
| Autism * Gender | .043 | 1 | | 0.32 | 0.5712 |
| **Comparison** | | | | | |
| NT Girls vs NT Boys | -.005 | .009 | -0.57 | 0.940 | -.029, .018 |
| Autistic Boys vs NT Boys | .044 | .030 | 1.48 | 0.451 | -.033, .122 |
| Autistic Girls vs NT Boys | .005 | .053 | 0.10 | 1.000 | -.131, .142 |
| Autistic Boys vs NT Girls | .050 | .030 | 1.65 | 0.353 | -.028, .127 |
| Autistic Girls vs NT Girls | .011 | .053 | 0.20 | 0.997 | -.125, .147 |
| Autistic Girls vs Autistic Boys | -.039 | .059 | -0.66 | 0.912 | -.191, .113 |
| **Unhappy with friends** (N = 7,514) | | | | | |
| Autism diagnosis | .543 | 1 | | 0.34 | 0.5616 |
| Gender (Female) | 1.717 | 1 | | 1.07 | 0.3021 |
| Autism * Gender | .435 | 1 | | 0.27 | 0.6034 |
| **Comparison** | | | | | |
| NT Girls vs NT Boys*** | .160 | .031 | 5.12 | 0.000 | .080, .241 |
| Autistic Boys vs NT Boys | .116 | .103 | 1.12 | 0.678 | -.150, .381 |
| Autistic Girls vs NT Boys | .169 | .183 | 0.92 | 0.793 | -.301, .639 |
| Autistic Boys vs NT Girls | -.045 | .104 | -0.43 | 0.973 | -.311, .221 |
| Autistic Girls vs NT Girls | .008 | .183 | 0.05 | 1.000 | -.461, .478 |
| Autistic Girls vs Autistic Boys | .053 | .204 | 0.26 | 0.994 | -.472, .578 |
